# Management of Choanal Atresia: National Recommendations with a Comprehensive Literature Review

**DOI:** 10.3390/children10010091

**Published:** 2023-01-02

**Authors:** Jure Urbančič, Domen Vozel, Saba Battelino, Imre Boršoš, Lev Bregant, Matic Glavan, Črtomir Iglič, Klemen Jenko, Boštjan Lanišnik, Tanja Soklič Košak

**Affiliations:** 1 Department of Otorhinolaryngology and Cervicofacial Surgery, University Medical Centre Ljubljana, 1000 Ljubljana, Slovenia; 2 Department of Otorhinolaryngology, Faculty of Medicine, University of Ljubljana, 1000 Ljubljana, Slovenia; 3 Division of Obstetrics and Gynecology, Department of Perinatology, Neonatal Intensive Care Unit, University Medical Center Ljubljana, 1000 Ljubljana, Slovenia; 4 Department of Otorhinolaryngology, Cervical and Maxillofacial Surgery, University Medical Centre Maribor, 2000 Maribor, Slovenia; 5 Department of Otorhinolaryngology, Faculty of Medicine, University of Maribor, 2000 Maribor, Slovenia

**Keywords:** nasopharynx, craniofacial abnormalities, infant, choanal atresia, charge syndrome

## Abstract

Choanal atresia is the most common congenital anatomical abnormality of the nasal cavities, manifested with a clinical picture of neonatal respiratory distress. The treatment requires interdisciplinary management based mainly on tertiary referral centre experiences. However, there is a lack of high-quality evidence in the available literature. Recommendations were prepared based on a systematic review of the supporting literature: on a website survey addressed to the participating authors consisting of 28 questions and on five live meetings. The initial response to the recommendations was determined at their presentation at the sectional meeting of the section for otorhinolaryngology of the Slovenian Medical Association. Then, reactions from the professional public were accepted until the recommendations were presented at the Expert Council for Otorhinolaryngology of the Slovenian Medical Association. A systematic literature review identified eight systematic reviews or meta-analyses and four randomized controlled clinical trials. Thirty-four recommendations for diagnosis, treatment and postoperative management were consolidated. The paper presents the proposal and first Slovenian recommendations for treating patients with choanal atresia. They are based on foreign medical institutions’ published literature and our clinical experience. They represent the basic requirements of diagnostics and may represent an essential guide in treatment.

## 1. Causes of Nasal Obstruction in an Infant

Normal nasal patency is essential for the breathing of a baby, who, in the first months of life, breathes mainly through the nose [1]. However, in the early period of a child’s life, breathing through the nose can be hindered due to congenital or acquired diseases of the nose or nasopharynx (Table 1). Congenital anatomical abnormalities of the nasal cavity (Table 1), including choanal atresia (CA), are rare causes of obstruction but often cause neonatal respiratory distress. In addition to congenital anatomical abnormalities, nasal obstruction can also be caused by congenital tumours, injuries, foreign bodies and inflammation. Inflammation of the nose and paranasal sinuses is the most common cause of nasal obstruction in infants but is mostly not directly life-threatening [2].

## 2. Epidemiology, Pathogenesis and Anatomical Characteristics of Choanal Atresia

Choanae are paired anatomical structures that are superiorly delimited by the inferior surface of the sphenoid body, medially by vomer, laterally by the medial plate of the pterygoid process and inferiorly by horizontal part of the palatine bone.

CA is the most common congenital anatomical abnormality of the nasal cavities [1]. It occurs in one in 5000–8000 neonates, twice as common in females, twice as often on the right and twice as often unilaterally (i.e., the rule of two) [4]. In 70%, CA is bony, and in 30%, it is bony-membranous since the atretic plate also consists of the membrane and the bone. Very rarely, CA is exclusively membranous [2]. The atretic plate may partially (i.e., stenosis) or completely (i.e., atresia) block the choana [4]. At least half of patients have syndromes associated with CA, most commonly CHARGE syndrome, characterized by coloboma (C), congenital heart defect (H), choanal atresia (A), mental retardation and growth disorder (R), developmental disorders of the genitals or urinary tract (G) and ear deformity (E) [5]. CA can occur as part of Treacher-Collins [2], Apert [2], fetal alcohol [2], Pfeiffer [2,6], Crouzon [4], Binder syndrome [2], ectrodactyly-ectodermal dysplasia clefting syndrome [7], hypoparathyroidism, deafness and renal dysplasia syndrome [8], mandibulofacial dysostosis [4], craniofacial clefts [9], etc.

CA results from disturbed embryogenesis in the fourth week of gestational age [2]. From the first description of the disease by the German physician Johann George Röderer in 1755 until today, the origin is explained with four theories: 1. persistence of the embryonic buccopharyngeal membrane, 2. persistence of the nasobuccal Hochstetter’s membrane, 3. improper migration of neural crest cells and 4. irregular growth of the mesoderm [10]. Disrupted embryogenesis is accompanied by medialization and ossification of the lateral nasal wall, especially the medial plates of pterygoid processes [2]. In addition, CA can occur due to vitamin A deficiency or taking thyrostatics during pregnancy [4].

## 3. The Clinical Picture of Choanal Atresia

The clinical picture of a patient with CA depends on the side and degree of impairment, associated craniofacial anomalies and systemic disease.

### 3.1. Bilateral Choanal Atresia

The position of the epiglottis above the soft palate and the tongue’s contact with the soft palate along its entire length a few months after birth prevents normal breathing through the mouth. The infant begins to learn to breathe through the mouth between the fourth and sixth week of age, allowing for normal feeding through the mouth [3]. At six months of age, mouth breathing is usually established [2]. As a result, bilateral CA due to nasal obstruction in the infant may occur as life-threatening respiratory distress immediately at birth, when the infant cannot breathe, or later as respiratory distress at first feeding.

The clinical picture of nasal obstruction consists of cyanosis during feeding and improvement of breathing during crying (i.e., cyclic cyanosis), episodes of apnea, snoring, nasal flutter, nasal congestion, nasal discharge, stertor, snoring, intercostal and jugulum retractions, hyponasal cry, aerophagia with abdominal tension, sleep disturbances and growth retardation [3]. In addition, in associated diseases of the head, neck or distant organs, when endotracheal intubation of the neonate is required immediately after birth, CA may first appear only after several failed extubation attempts [4].

Bilateral CA is associated in 34% with other upper respiratory tract abnormalities, which lead to respiratory distress. These include subglottic stenosis, laryngomalacia and tracheomalacia. In addition, in 21%, bilateral CA is accompanied by other congenital craniofacial abnormalities (Table 1) [5].

### 3.2. Unilateral Choanal Atresia

Unilateral CA rarely causes neonatal respiratory distress syndrome. It is most commonly expressed later than bilateral CA, with a clinical picture of chronic unilateral nasal discharge and unilateral obstruction, which may resemble chronic rhinosinusitis or a foreign body in the nasal cavity. As a result, unilateral CA is often diagnosed later in adulthood [4].

## 4. Materials and Methods

When preparing the recommendations, we followed the procedure of Rosenfeld et al. (2013) for the preparation of clinical guidelines and recommendations in otorhinolaryngology—head and neck surgery [11] and general instructions for the preparation of clinical guidelines [12].

Instead of conference calls, five live meetings were held. Recommendations were written (Table 2), which were coordinated with the participating authors and institutions based on the supporting literature searched according to the procedure described in Table 3 and a website survey addressed to the participating authors consisting of 28 questions following the example of Moreddu et al. (2019) [13]. In addition, eight systematic reviews or meta-analyses [14,15,16,17,18,19,20,21] and four randomized controlled clinical trials [22,23,24,25] were identified.

For each recommendation, we determined the level of evidence and the evidence grade using the OCEBM Levels of Evidence (Table 4) [29]. In addition, we examined the risks and benefits of adopting each recommendation. Finally, we determined four levels of strength of recommendations (i.e., four types of text usage) following the example of the recommendations of the American Academy of Otolaryngology-Head and Neck Surgery (Table 4): strongly recommended, recommended, optional and strongly recommended against [11]:

∙ Strongly recommended means that the physician should follow the recommendation unless there is a clear and compelling argument against the recommendation.

∙ Recommended means that the physician should follow the recommendation but pay attention to new information and patient peculiarities.

∙ Optional means that the physician must be flexible in making decisions and accepting the recommendation. He must consider the possibility of taking different measures and measures adapted to the patient [11].

First, the evidence grade was determined for the included studies according to OCEBM [29], and then, the determination of strength with the benefit–harm ratio assessment by Rosenfeld et al. (2013) [11].

The preliminary response to the recommendations was determined at their presentation at the sectional meeting of the section for otorhinolaryngology of the Slovenian Medical Association on 19th December 2020. We accepted responses from the professional public until the recommendations were presented at the Expert Council for Otorhinolaryngology of the Slovenian Medical Association. This is the umbrella regulatory body for the field of otorhinolaryngology in Slovenia. It reviewed recommendations, suggested corrections and approved them on 10th May 2021.

## 5. Results

Thirty-four recommendations were written (Table 2) and coordinated based on the website survey and review of the literature (Table 3), which identified eight systematic reviews or meta-analyses [14,15,16,17,18,19,20,21] and four randomized controlled clinical trials [22,23,24,25].

### 5.1. Diagnosis of Choanal Atresia

#### 5.1.1. Bilateral Choanal Atresia

At birth or in the early postpartum period, the clinical picture of respiratory obstruction should be checked for nasal patency to rule out bilateral CA [30] and other causes of nasal obstruction (Table 1).

**Figure 1:**
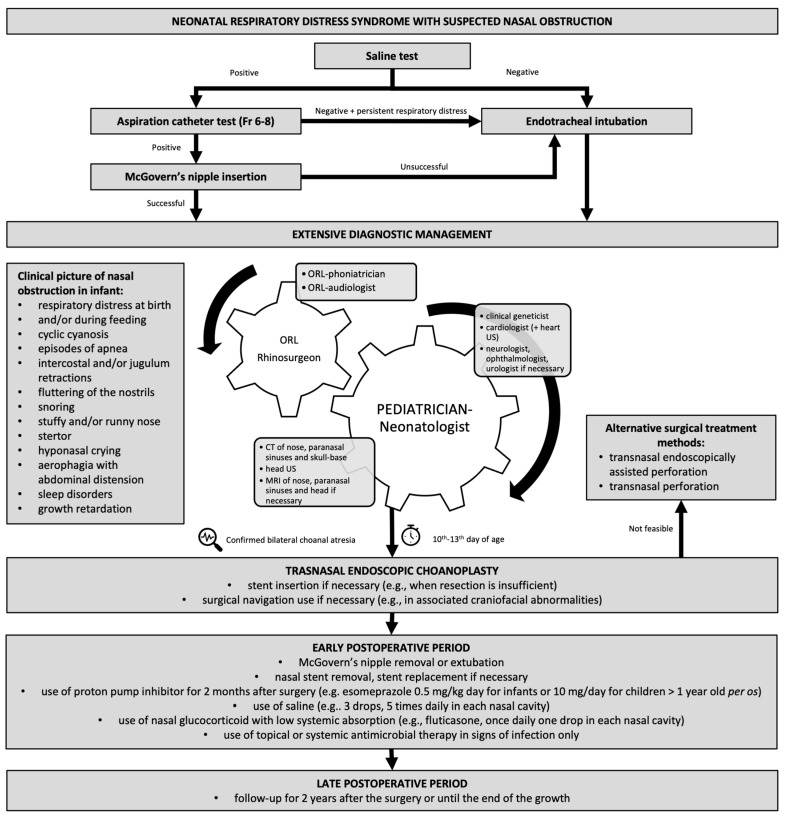
Algorithm for neonatal respiratory distress syndrome due to nasal obstruction caused by bilateral choanal atresia. The paediatrician and otorhinolaryngologist play a leading role in extensive diagnostic management. In cooperation with the otorhinolaryngologist, the paediatrician also takes care of coordination in treating the child by other specialists and for imaging and diagnostic evaluation. The otorhinolaryngologist primarily provides management by other subspecialist otorhinolaryngologists. A positive saline and aspiration catheter test means that the saline or catheter does not pass through the nasal cavity into the pharynx.

**Recommendation** **1:** *In the case of respiratory distress in a neonate, we recommend that the physician perform a test with a saline solution to confirm or rule out nasal obstruction—level of Evidence IIa, evidence grade C (Table 2) (Figure 1).*

A few drops of saline solution are instilled into both nostrils. If the saline solution pours forward from the nostrils, the test is positive on the side (or both) where the spill is observed. This makes CA likely. If the saline solution passes into the throat, the test is negative. Therefore, CA is not possible, but respiratory distress can be caused by other causes of nasal obstruction (Table 1).

**Recommendation** **2:** *We recommend a positive saline test followed by a Fr 6-8 aspiration catheter test—level of Evidence IIa, evidence grade C (Table 2) (Figure 1).*

An aspiration catheter is used to assess the patency of the nasal cavities and the nasopharynx and, in some cases, eliminates the reversible cause of nasal obstruction (e.g., meconium plug, lanugo, vernix). If it is not possible to insert the aspiration catheter 1–2 cm beyond the nostril, the obstruction is most likely due to deviation of the nasal septum or thickening of the inferior nasal turbinate, and in the case of the obstruction 3–3.5 cm deep, it is most likely CA or choanal stenosis. In addition to the test with an aspiration catheter, there is also a test with the instillation of methylene blue in the nostril with the examination of the pharynx, a test with a cotton swab or a mirror to observe the airflow through the nostrils [4] and the use of a stethoscope with the funnel removed to listen to the airflow through the nostrils [31].

After establishing a free airway and the initial clinical examination, an extended diagnostic workup is required in the early postpartum period, which depends on the clinical presentation and risk factors for associated abnormalities.

**Figure 2:**
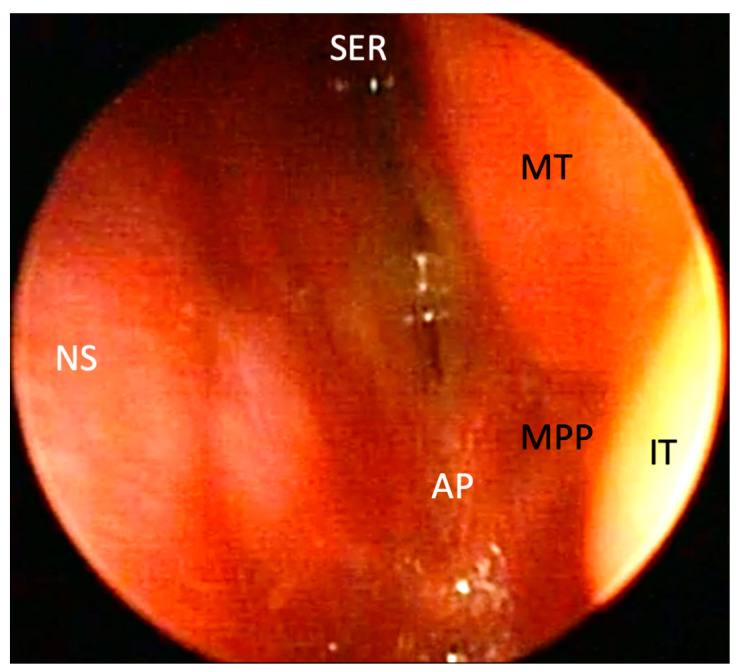
Endoscopic photograph of the left nasal cavity in a three-day-old neonate with bilateral bony choanal atresia. SER—sphenoethmoid recess; NP—nasal septum; MT—middle turbinate; IT—inferior turbinate; MPP—medial pterygoid plate; AP—atretic plate.

**Recommendation** **3:** *We strongly recommend that the otorhinolaryngological examination include examining the nose and face to rule out associated abnormalities (Table 1). This should be followed by anterior rhinoscopy and toilet, anemization, and epimucosal anaesthesia of the nasal cavities with the selected anaesthetic. An examination should then be performed with a flexible or rigid endoscope—level of evidence I, evidence grade A (Figure 2) (Table 2) [13].*

**Recommendation** **4:** *Although CA is a clinical diagnosis, we strongly recommend that a 2–5 mm slice thickness CT scan of the facial structures, nose, paranasal sinuses and skull base be performed in every patient to confirm the diagnosis and plan treatment—level of evidence I, evidence grade A (Table 2) [4,13].*

**Figure 3:**
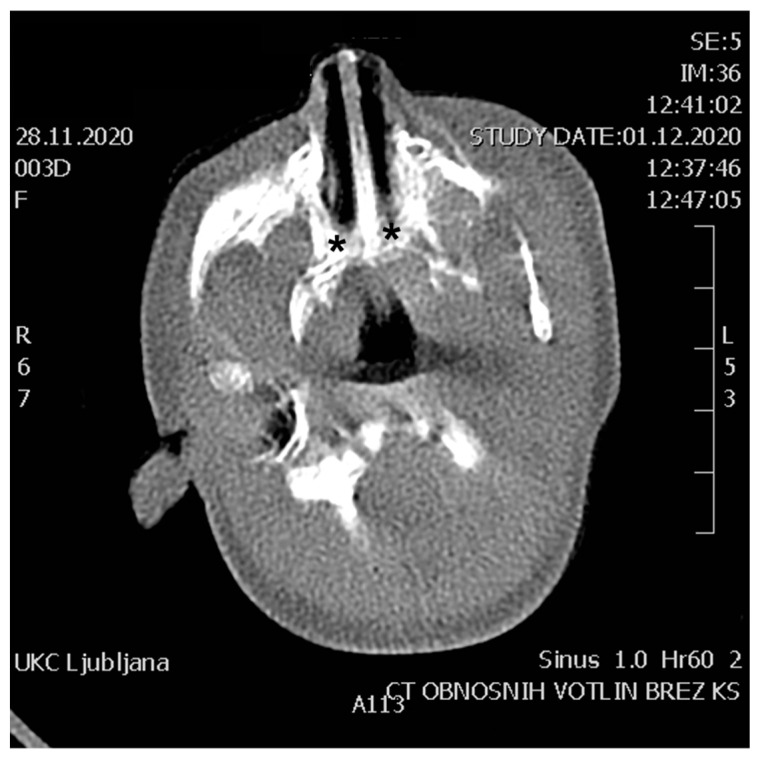
CT of the nose and paranasal sinuses in the axial plane. Bilateral bony choanal atresia is seen in a three-day-old neonate. * indicates atretic plate.

A CT can also identify other causes of nasal obstruction (Table 1) [4]. Before CT imaging, it is necessary to aspirate both nostrils, as mucus can be interpreted as soft tissue abnormalities, for example, encephalocele [32]. On CT, bony thickening of the medial plates of the pterygoid processes and thickening of the posterior part of the vomer is usually visible, narrowing or filling the lumen of the choana (Figure 3) [4]. At the same time, the same examination can also evaluate associated anatomical abnormalities, especially otological [13]. If the semicircular canal abnormalities are found simultaneously, CHARGE syndrome is very likely.

**Recommendation** **5:** *We strongly recommend that a head ultrasound be performed to rule out central nervous system involvement, which is common, especially in preterm newborns—level of Evidence I, evidence grade C (Table 2) [26].*

An MRI of the head should only be performed in CA in selected cases [33].

**Recommendation** **6:** *Bilateral CA often occurs in the context of other congenital syndromes, so clinical geneticist consultation is strongly recommended—level of Evidence I, evidence grade B (Table 2).*

**Recommendation** **7:** *From the point of view of the need for surgical care of a child with bilateral CA, we strongly recommend excluding congenital abnormalities that would put the child at risk during the procedure, especially heart disease, so we recommend an evaluation by a cardiologist—level of Evidence I, evidence grade B (Table 2).*

**Recommendation** **8:** *Even within otorhinolaryngology and in the postoperative period, we strongly recommend an audiovestibulological examination for a hearing evaluation and evaluation by a phoniatrician for additional swallowing problems—level of evidence I, evidence grade D (Table 2) [2,4,5,7,8,9].*

A neurologist, urologist or ophthalmologist is included in the treatment if necessary (Figure 1) [13].

#### 5.1.2. Unilateral Choanal Atresia

In the case of unilateral CA, the diagnosis is usually established later, as the clinical picture of unilateral CA is rarely manifested as respiratory distress. Therefore, diagnostics can be performed on an outpatient basis. The otorhinolaryngologist determines the patency of the nose using an aspiration catheter (appropriate size for the patient’s age), anterior rhinoscopy and nasal endoscopy. Despite everything, he must also pay special attention to possible associated diseases and refer the patient for further treatment.

**Recommendation** **9:** *In the case of unilateral CA, we recommend a CT of the facial structures, nose, paranasal cavities and skull base be performed. With an unmistakable clinical picture and endoscopic status, this is indicated only before surgical treatment to avoid the unnecessary exposure of the child to ionizing radiation—level of Evidence IIa, evidence grade D (Table 2).*

**Recommendation** **10:** *In all cases of unilateral CA, we strongly recommend audiovestibulological evaluation, and for additional swallowing problems, treatment by a phoniatrician—level of evidence I, evidence grade D (Table 2).*

### 5.2. Treatment

#### 5.2.1. Bilateral Choanal Atresia

##### Airway Management

Before a thorough diagnosis and therapy, it is necessary to immediately ensure a free airway through the mouth by inserting a McGovern nipple in case of bilateral CA (Figure 1). Then, a nasogastric or feeding tube is inserted next to or through the nipple. An endotracheal intubation is required if a free airway cannot be established with a nipple [4]. In any case, the definitive treatment of the airway in a child who breathes on his own is to ensure the patency of the nose as soon as possible. Tracheotomy is considered only if long-term mechanical ventilation is expected in the infant, for example, with associated cardiac, pulmonary, neurological disease or multilevel respiratory obstruction. More specifically, the indications for tracheotomy were described by Walsh et al. (2018) [34]. If possible, tracheotomy is not recommended in cases where successful surgical treatment of CA can be performed. This paper does not provide an opinion or recommendations about tracheostomy.

##### Surgical Treatment

**Recommendation** **11:** *We strongly recommend that bilateral CA be surgically treated between the 10th and 13th day of the neonate’s age, even in the case of prematurity—level of evidence I, evidence grade A (Table 2) [16,27].*

Before surgical treatment, it is first necessary to carry out urgent diagnostic procedures to determine associated diseases and risk factors, especially cardiological treatment (Figure 1) [13]. Then, if the procedure under general anaesthesia is safe, the otorhinolaryngologist rhinosurgeon, will decide on the timing of the surgical treatment.

Even though the first description of surgical treatment of CA dates back to the mid-19th century, there is still no clear consensus on surgical techniques to date [4]. There are not enough systematic reviews and meta-analyses; only one systematic literature review was published in the Cochrane Library, in which it was found that out of 46 reviewed studies with descriptions of surgical techniques, none were suitable for the final analysis [14].

**Figure 4:**
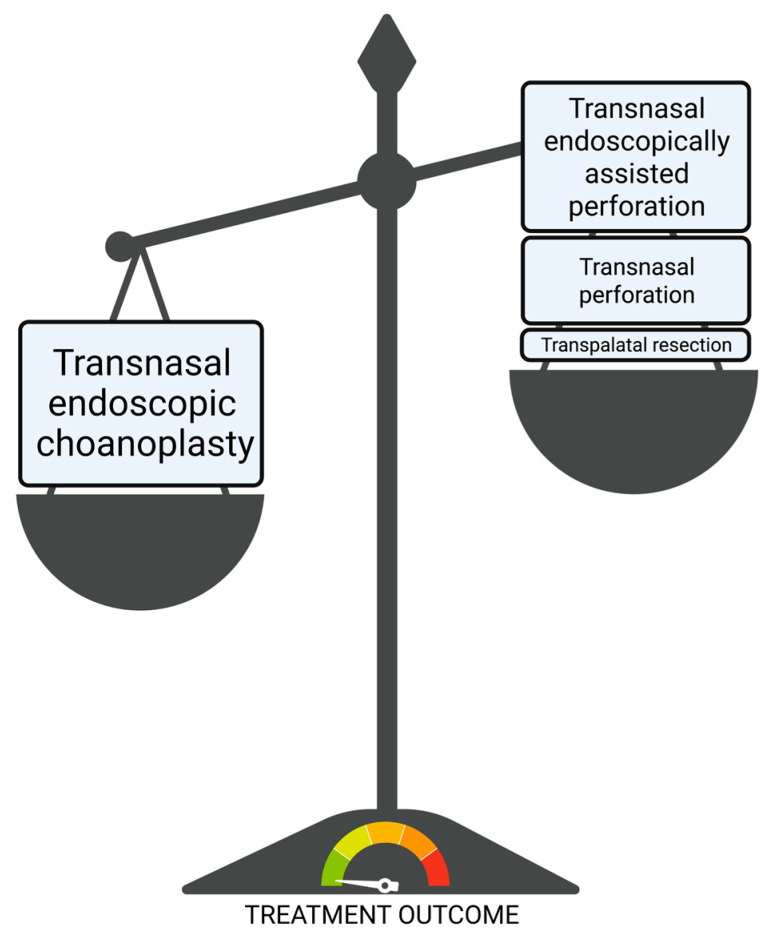
Types of surgical treatment methods of choanal atresia. Transnasal endoscopic choanoplasty is the gold standard with the best treatment outcome and lowest restenosis rate. Other surgical techniques present an alternative, e.g., transnasal endoscopically controlled perforation in cases where the manipulation with instruments is impossible due to the size of the nasal cavities, for example, in preterm infants. Transnasal perforation and transpalatinal resection represent the hierarchical bottom of surgical techniques and should not be performed.

**Figure 5:**
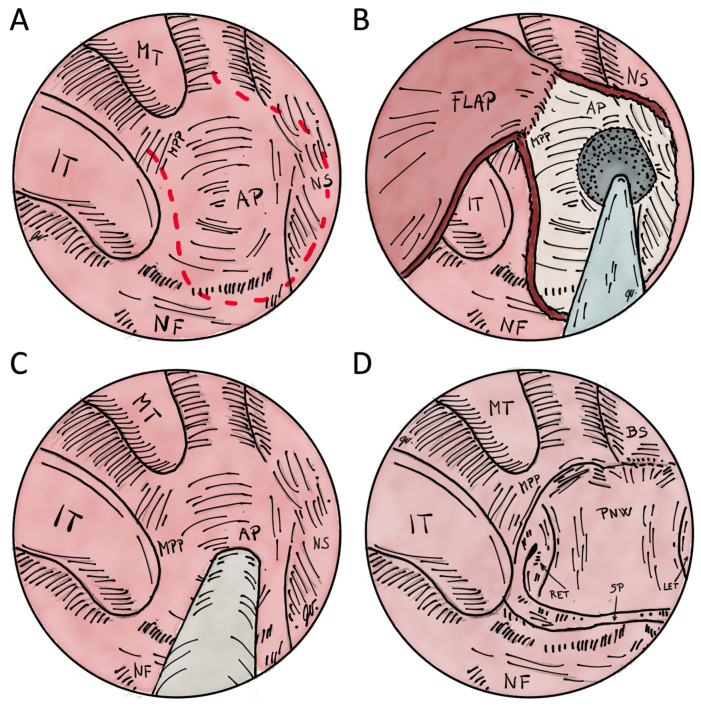
Illustrations of transnasal endoscopic choanoplasty and perforation for the treatment of choanal atresia. (**A**)–red dashed line shows flap margins before resection of atretic plate in right nasal cavity. (**B**)–flap is elevated to drill the exposed bone of the atretic plate and posterior nasal septum resection. After the resection, the flap is layed on the exposed bone of medial pterygoid plate and base of the sphenoid. (**C**)–transnasal perforation technique is shown with or without the assistance of endoscope. There is no mucosal flap elevation. (**D**) after posterior nasal septum resection and drilling of atretic plate to the level of medial pterygoid plate, an unichoana is created. MT–middle turbinate; IT–inferior turbinate; AP–atretic plate; NS–nasal septum; NF–nasal floor; MPP–medial pterygoid plate; BS–base of the sphenoid sinus; RET–right Eustachian tube; LET–left Eustachian tube; PNW–posterior nasopharyngeal wall; SP–soft palate.

**Figure 6:**
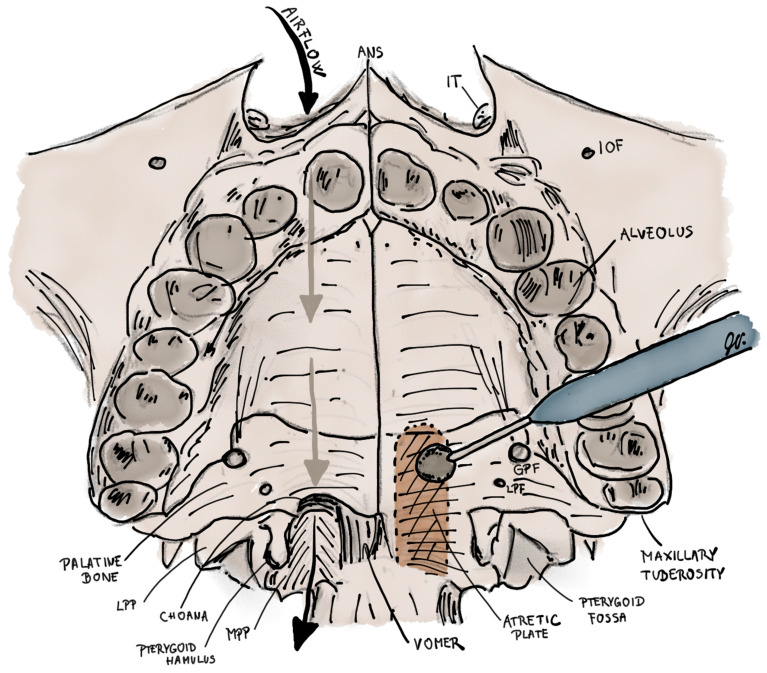
Illustration of transpalatal resection for the treatment of left-sided choanal atresia. Right nasal cavity has a normal patency. First the palatal flap pedicled posteriorly on both greater palatine arteries is elevated to expose the bone of hard palate. Then, the atretic plate, which is located between vomer and medial pterygoid plate, is burred (coloured brown) up to the height to the base of sphenoid sinus, medially to the vomer and laterally to the level of medial pterygoid plate. ANS–anterior nasal spine; IT–inferior turbinate; IOF–infraorbital foramen; LPP–lateral pterygoid plate; MPP–medial pterygoid plate; GPF–greater palatine foramen; LPF–lesser palatine foramen.

Surgical treatment can be divided into transnasal perforation, transpalatal resection, transnasal endoscopically assisted perforation and transnasal endoscopic choanoplasty (Figure 4). A stent can also be inserted regardless of the method to maintain choanal patency [4]. All surgical techniques are illustrated in Figure 5 and Figure 6.

##### Transnasal Endoscopic Choanoplasty

**Figure 7:**
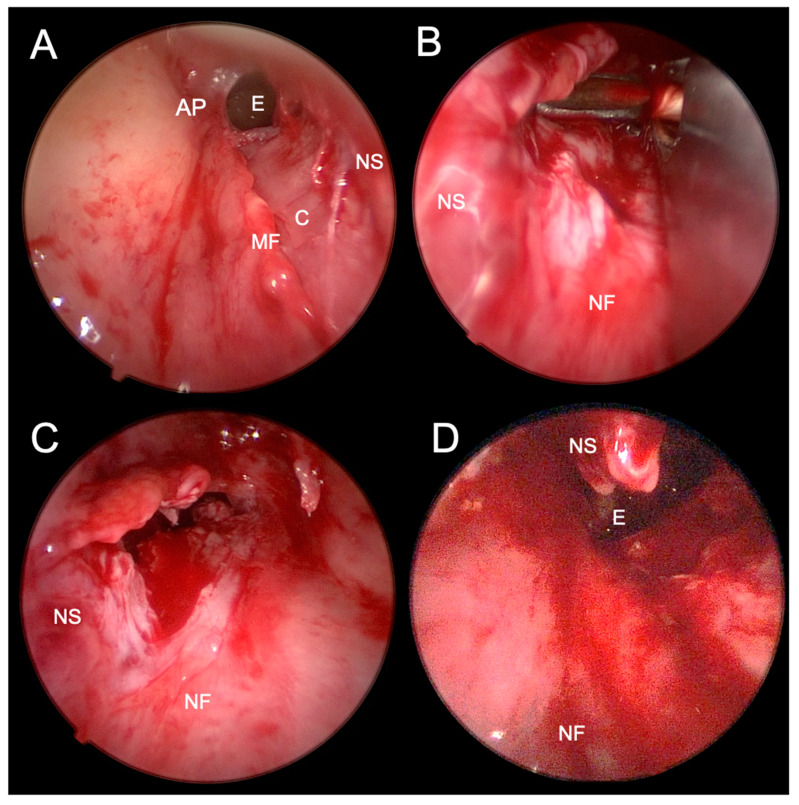
Endoscopic photographs of transnasal endoscopic choanoplasty of bilateral choanal atresia. (**A**)–after perforation of the atretic plate of the right nasal cavity and elevation of the mucoperichondrial flap of the nasal septum and atretic plate. (**B**)–resection of the posterior nasal septum with forceps through the left nasal cavity. (**C**)–after partial resection of the posterior nasal septum visible through the left nasal cavity. (**D**)–after a partially surgically formed unichoana, which connects both nasal cavities in the posterior nasal septum resection area. AP–atretic plate; E–epipharynx; C–cartilage; MF–mucosal flap; NS–nasal septum; NF–nasal floor.

Transnasal endoscopic choanoplasty is considered the surgical technique of choice for the treatment of CA by most otorhinolaryngologists [4]. It is based on the endoscopic removal of the atretic area and the posterior part of the nasal septum, thereby creating a single choana (i.e., unichoana) (Figure 5A,B,D). Instruments for cold steel bone resection are primarily used, and, in the case of medialized medial pterygoid plates, a diamond drill is used [13] (Figure 7). The successful use of the CO_2_ laser [13,35] and balloon dilators [13,36] has also been described. Suppose it is impossible to manipulate the instrument through the same nostril; the endoscope is inserted in the contralateral nasal cavity. In that case, it is advised to first puncture the posterior part of the nasal septum under the control of the endoscope and then continue resectioning the atretic plate with the endoscope inserted in the contralateral nasal cavity. The mucosa must be preserved in the form of flaps, which are placed on the exposed bony walls of the neochoana at the end of the procedure, thereby preventing restenosis [4,20]. The success rate of transnasal endoscopic choanoplasty, determined by the occurrence of restenosis or the need for revision, is 65% according to a meta-analysis by Strychowsky et al. (2015) [18]. The possible risk factors for restenosis are associated congenital abnormalities, reflux of gastric contents in the nasopharynx and the age of the neonate <10 days since a lower age determines more unfavourable anatomical conditions that limit the visualization and extent of resection [4].

**Recommendation** **12:** *Transnasal endoscopic choanoplasty is strongly recommended in all patients with bilateral CA in whom an endoscopic approach is possible—level of evidence I, evidence grade A (Figure 1) (Table 2) [13].*

The insertion of stents, taking into account the advantages and disadvantages, can neither be recommended nor advised against since the success rate, regardless of stent insertion, is only 65% [18]. The advantages of using stents are a lower incidence of restenosis and satisfactory patency in the initial postoperative period. At the same time, the disadvantages are the need for more frequent treatment due to stent changes, irritation, erosion or ulceration of the nasal cavity, which can lead to the formation of adhesions and restenosis [4,21].

**Recommendation** **13:** *If a stent is used, we recommend its removal within seven days to reduce the risk of complications [18] —level of Evidence IIa, evidence grade A (Table 2).*

**Recommendation** **14:** *In case of associated craniofacial abnormalities, we recommend using navigation, which can be CT and/or MR-guided—level of evidence IIa, evidence grade C (Table 2).*

Navigation enables easier recognition of altered anatomical conditions and safer intervention in CA [4,9].

##### Transnasal Perforation

Transnasal perforation is an older surgical technique primarily performed blindly with a probe or dilator through the nostrils. Later, they started to simultaneously use a 120° endoscope or a mirror to examine the area of atresia. However, especially with the blind technique, there is a significant risk of complications arising from damage to the nasal septum, lateral nasal wall, nasal vault or clivus [4]. In addition, a high risk of restenosis has also been described [10].

**Recommendation** **15:** *Transnasal perforation with stent insertion is allowed as an option in patients with thin bony-membranous CA or stenosis and in patients in whom transnasal endoscopic perforation is not possible due to small nasal lumen—level of evidence IIb, evidence grade C (Figure 1) (Table 2) [4].*

A stent can be a shortened endotracheal tube, an aspiration catheter or a similar medical device [25]. All should be changed regularly postoperatively (described below). In addition, the effective use of corticosteroid-eluting stents has also been described [37].

The use of mitomycin has been described in the prevention of restenosis. Still, it is not recommended due to its potential carcinogenicity and the lack of clinical efficacy data so far [13].

##### Transnasal Endoscopically Assisted Perforation

**Recommendation** **16:** *Transnasal endoscopically assisted perforation with stent insertion is allowed as an option when transnasal endoscopic choanoplasty is not possible due to anatomical conditions—level of evidence IIb, evidence grade C (Table 2) [4,13].*

The procedure is identical to the described blind perforation with additional endoscopic control. This reduces the possibility of unwanted damage to adjacent tissues (Figure 1 and Figure 5C).

In the case of prematurity, endoscopic transparency and the use of endoscopic instruments are limited, so transnasal endoscopic choanoplasty is often not possible. Then, transnasal endoscopically assisted perforation is the method of choice for treating bilateral CA. This procedure will most likely require a later (so-called revision) transnasal endoscopic choanoplasty when the child has grown, and the anatomical conditions allow this surgical treatment method.

There is no mucosal flap elevation and posterior nasal septum resection in transnasal perforation techniques (endoscopically assisted or unassisted).

##### Transpalatal Resection

In transpalatal resection, the mucous membrane of the hard palate is raised in a local flap, and the entire thickness of the bone in the area of bony atresia is drilled away (Figure 6). Despite the low incidence of restenosis, the complications of this operation are significant. These are malocclusion, palate necrosis, oronasal fistula, soft palate muscle dysfunction and velopharyngeal insufficiency. Therefore, this method is not recommended for children under six.

**Recommendation** **17:** *In the primary treatment of CA, we recommend against transpalatal resection of CA—level of evidence III, evidence grade A (Table 2) [4,13].*

#### 5.2.2. Unilateral Choanal Atresia

The age at which unilateral CA is treated depends on the patient’s age at the time of the unequivocal diagnosis, which is based on the results of a systematic review by Murray et al. (2019).

**Recommendation** **18:** *Surgical treatment of unilateral CA is recommended after the infant is six months old—level of evidence IIa, evidence grade A (Figure 1) (Table 2) [13].*

Even with the start of treatment after the third year of age, we expect the same results as before [13].

**Recommendation** **19:** *Transnasal endoscopic choanoplasty is strongly recommended for unilateral CA due to the mostly good endoscopic visualization in older patients with unilateral CA. The extent of the resection should be large enough so that it is not necessary to use a stent—level of evidence I, evidence grade A (Table 2) [13].*

### 5.3. Postoperative Management

Postoperative management depends on the CA’s location, the patient’s general health and the patient’s setting (i.e., outpatient or inpatient).

#### 5.3.1. Bilateral Choanal Atresia

Postoperative treatment after surgical treatment of bilateral CA depends mainly on associated diseases, the gestational age of the neonate and the related need for treatment of other conditions. Children with CHARGE syndrome have a higher risk of postoperative complications and prolonged hospitalization [27]. The same is to be expected in premature infants. The intubated patient has an additional chance of complications due to extended postoperative mechanical ventilation and prolonged hospitalization [38].

##### Stented

**Recommendation** **20:** *In the case of bilateral CA where a stent was used to ensure nasal patency, it is recommended that the stent be changed several times a week endoscopically during the first and second postoperative week—level of evidence IIa, evidence grade D (Figure 1) (Table 2).*

During this period, it is reasonable to increase the outer diameter of the stent so that the lumen of the neochoana increases significantly, such as twice the outer diameter of the endotracheal tube, as long as the anatomical conditions allow this increase (we did not reach the maximum possible dimensions). This is especially necessary after transnasal endoscopically guided perforation and transnasal perforation. After transnasal endoscopic choanoplasty, the insertion of stents is often not needed.

**Recommendation** **21:** *We recommend that discharge from the hospital be planned for the second postoperative week or as soon as possible when the general state of health allows it and the patency of the stents is satisfactory with appropriate care—level of Evidence IIa, evidence grade D (Table 2).*

We monitor the child’s breathing patterns until discharge. In addition to normal breathing, the main goal of treatment for bilateral CA is independent feeding without breaks as soon as possible after surgical treatment.

**Recommendation** **22:** *Stent replacement on an outpatient basis in the second week is optional—level of evidence IIb, evidence grade D (Table 2).*

**Recommendation** **23:** *In the third week, it is optional to replace the stent twice, and from the fourth week onwards, only once more—level of evidence IIb, evidence grade D (Table 2).*

**Recommendation** **24:** *We strongly recommend removing the stent in the coming weeks and rechecking the patency the week after—level of Evidence I, evidence grade D (Table 2).*

The replacement of stents in the postoperative period can be postponed as long as their patency is satisfactory. Instead of changing stents, we can perform regular dilations, especially after transnasal endoscopically controlled perforation and transnasal perforation, for example, with a balloon dilator, which has already been used in the treatment of bilateral CA [36].

**Recommendation** **25:** *Photo documentation of the lumen is recommended—level of evidence IIa, evidence grade D (Table 2).*

The intervals between check-ups are gradually being prolonged. Otherwise, restenosis after one year is rare [13].

**Figure 8:**
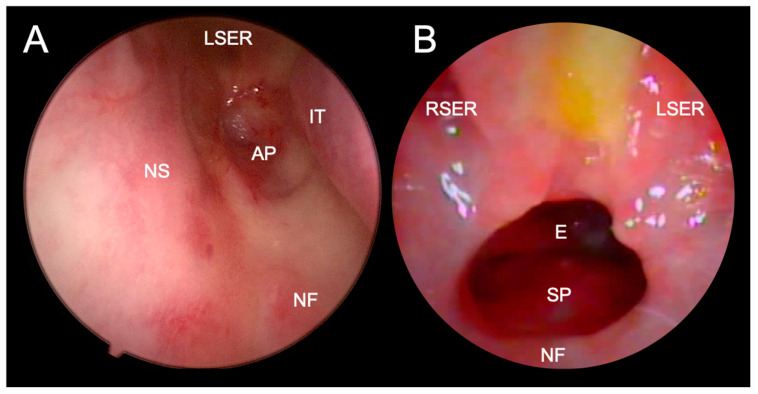
Endoscopic visualization of complete restenosis after transnasal perforation (**A**) and good patency of unichoana four weeks after transnasal endoscopic choanoplasty (**B**) of bilateral choanal atresia. A trace of milk and mucus is visible between the two sphenoethmoid recesses in Figure B, suggesting a significant influence of gastric contents reflux on the operative wound healing. LSER - left sphenoethmoid recess; RSER–right sphenoethmoid recess; IT–inferior turbinate; AP–atretic plate; NS–nasal septum; NF–nasal floor; E–epipharynx; SP–soft palate.

**Recommendation** **26:** *Follow-up is strongly recommended for at least two years after the procedure or until the end of growth to detect restenosis—level of evidence I, evidence grade C (Figure 1 and Figure 8) (Table 2).*

Upon discharge from the hospital, it is necessary to include the child’s parents in the treatment process. They should be taught how to clean the nose effectively and reinsert the stent, and clear instructions should be given in the event of the infant’s respiratory distress. In the case of breathing problems, poor nasal patency or discharge, a repeat examination by an otorhinolaryngologist is necessary to assess choanal patency accurately. In the postoperative period, we plan as few examinations as possible in the hospital.

**Recommendation** **27:** *Revision transnasal endoscopic surgery is recommended in case of breathing problems, poor nasal patency or discharge and more than 50% reduction of the choanal lumen—level of evidence IIa, evidence grade A (Table 2).*

CT or other radiological examinations are not routinely indicated in postoperative management, even in restenosis [13].

**Recommendation** **28:** *During the postoperative treatment of the infant, the daily use of proton pump inhibitors is recommended for the first two months (e.g., per os esomeprazole 0.5 mg/kg day for infants or 10 mg/day for children over one year of age)—level of evidence IIa, evidence grade C [25].*

**Recommendation** **29:** *During the postoperative management of the infant, the instillation of saline solution (e.g., 3 drops, 5 times a day in each nostril) and nasal glucocorticoid drops with low systemic absorption (e.g., fluticasone, one time a day, one drop in each nostril) are strongly recommended for the first two months—level of evidence I, evidence grade C [13,28].*

The drops should be instilled next to the stent and not into it.

**Recommendation** **30:** *During the first two months of postoperative treatment of the infant, antibiotic drops are recommended exceptionally in the first postoperative week or later when noticeable purulent discharge appears—level of Evidence IIa, evidence grade D (Table 2).*

In the case of systemic signs of infection, identification of the source of infection and systemic treatment are indicated.

##### Non-Stented

In the postoperative treatment of patients after transnasal endoscopic choanoplasty of bilateral CA without inserted stents, the infant can already start eating food by mouth on the first day, as soon as the effects of general anaesthesia (anaesthetics and narcotics) wear off after a few hours. We monitor the child’s breathing patterns, which must be appropriate in all life circumstances (sleeping and feeding). Otherwise, it is necessary to determine the cause of breathing problems, which may be local or an associated, unrecognized pathology.

**Recommendation** **31:** *The first postoperative examination with the assessment of nasal breathing with the mouth closed and feeding by mouth without pauses for breathing is recommended the day after surgery (first postoperative day)—level of evidence Iia, evidence grade D.*

**Recommendation** **32:** *We allow the possibility of discharge from the hospital on the second postoperative day—level of evidence Iib, evidence grade D.*

**Recommendation** **33:** *We recommend nasal endoscopy one week after surgery, two weeks after surgery and four weeks after surgery—level of evidence IIa, evidence grade D (Figure 1) (Table 2).*

Photo documentation enables comparison between inspections. Instructions to parents and recommendations regarding the use of drops and revision surgery are the same as for patients with inserted stents [13].

#### 5.3.2. Unilateral Choanal Atresia

**Recommendation** **34:** *With unilateral CA, the risk of respiratory distress is very low, so we recommend discharge on the first postoperative day—level of evidence IIa, evidence grade D (Table 2).*

We perform the first examination on this day, especially the nasal cavity toilet. We are planning the subsequent outpatient check-up in one week. In the following weeks, the otorhinolaryngologist decides on the frequency of check-ups. Parental instructions and recommendations regarding the use of drops, follow-up and revision surgery are the same as for patients with stents.

## 6. Conclusions

The paper presents the first Slovenian recommendations for treating patients with choanal atresia. They are based on foreign medical institutions’ published literature and our clinical experience. They represent the basic requirements of diagnostics and are a possible essential guide in treatment, which, however, must be adapted according to the current situation. Therefore, a thorough review of each recommendation is necessary before implementation.

However, these recommendations focus on the otorhinolaryngological management of choanal atresia, which should be considered. Moreover, only a small number of systematic reviews and meta-analyses are included in this study.

In further decades of experience and technology development, recommendations can be expected to improve due to changes in treatment, especially transnasal endoscopic surgical techniques, the use of stents and other methods of preventing restenosis. Therefore, it makes sense to create a register of patients with choanal atresia and other congenital anomalies of the craniofacial area, upper respiratory tract and gastrointestinal tract for prospective data collection.

## Figures and Tables

**Table 1:** Causes of nasal obstruction in infants.

Congenital tumours	● Neoplasms: chordoma, teratoma, haemangioma, rhabdomyosarcoma, craniopharyngioma, hamartoma, histiocytoma, hemangiopericytoma, lipoma, vascular malformation, congenital squamous cell carcinoma, teratoid, lymphoma ● Cysts: dacryocystocele, incisive canal cyst, dentigerous cyst, mucocele, Thornwaldt’s cyst ● Midline masses: nasal dermoid, glioma, meningoencephalocele, encephalocele
Congenital anatomical abnormalities	● Nasal agenesis or partial dysgenesis ● Anterior nasal stenosis ● Midline nasal stenosis ● Pyriform aperture stenosis ● Choanal atresia/stenosis ● Craniofacial syndromes: CHARGE, Binder, Crouzon, Apert, Treacher-Collins, Pfeiffer, Kabuki
Traumatic	● Nasal tip depression ● Iatrogenic stenosis ● Nasal septum fracture/dislocation ● Nasal septum haematoma/abscess ● Foreign body
Inflammatory	● *Rhinitis medicamentosa*: mother’s or infant’s medications ● Neonatal rhinitis: meconium, allergy, GER, nasal milk regurgitation, infections (*Staphylococcus aureus, Chlamydia trachomatis, Treponema pallidum,* RSV etc.)
Metabolic diseases	● Hypothyroidism

Table 1 adapted from Roehm et al. (2013) and Gnagi et al. (2013) [2,3]. CHARGE—a syndrome characterized by coloboma (C), congenital heart defect (H), choanal atresia (A), mental and growth retardation (R), genitourinary tract (G) and ear abnormalities (E); GER—gastroesophageal reflux; RSV–respiratory syncytial virus.

**Table 2:** Summary of Slovenian recommendations for the management of choanal atresia.

Nr	Recommendation	L	Gr	Strength
1,2	In the case of respiratory distress in a neonate, a saline test should be performed to confirm nasal obstruction, followed by a test with an aspiration catheter of size Fr 6–8 [13]	IIa	C	Recommended
3	The otorhinolaryngological examination should include examination of the nose and face, anterior rhinoscopy, toilet, anemization, epimucosal anaesthesia of the nose and examination with a flexible or rigid endoscope [13].	I	A	Strongly recommended
4	In the case of bilateral CA, CT of the facial structures, nose, paranasal cavities and skull base should be performed with a slice thickness of 2–5 mm [4,13]	I	A	Strongly recommended
5	Head ultrasound should be performed for bilateral CA [26]	I	C	Strongly recommended
9	In the case of unilateral CA, a CT of the facial structures, nose, paranasal cavities, and skull base should be performed with a slice thickness of 2–5 mm [13]	IIa	D	Recommended
6, 7	In the case of bilateral CA, congenital abnormalities should be ruled out, so a clinical geneticist and a cardiologist should be involved because of heart diseases that would put the child at risk during the procedure [13]	I	B	Strongly recommended
8, 10	In the case of unilateral or bilateral CA, an audiovestibulological evaluation should be performed to assess hearing and treatment by a phoniatrician in case of additional swallowing problems [13]	I	D	Strongly recommended
11	Bilateral CA should be surgically treated between the 10th and 13th day of the neonate’s age, even in the case of prematurity (Murray et al. 2019; Marston et al. 2019) [16,27]	I	A	Strongly recommended
12	Transnasal endoscopic choanoplasty should be performed in all patients with bilateral CA in whom an endoscopic approach is possible [13]	I	A	Strongly recommended
13	If a stent is used, it should be removed within seven days [18]	IIa	A	Recommended
14	In case of associated craniofacial abnormalities, neuronavigation should be used [4,9]	IIa	C	Recommended
15	Transnasal perforation with stent insertion can be performed in patients with thin bony-membranous bilateral CA or stenosis and in patients in whom transnasal endoscopically assisted perforation is not possible due to insufficient nasal lumen [4]	IIb	D	Optional
16	Transnasal endoscopically assisted perforation with stent insertion can be performed when transnasal endoscopic choanoplasty of bilateral CA is not possible due to anatomical conditions [4,13]	IIb	C	Optional
17	Transpalatal resection should not be the primary method of treatment for unilateral or bilateral CA [4,13]	III	A	Recommended against
18	Surgical treatment of unilateral CA should be performed after the baby is six months old [13]	IIa	A	Recommended
19	Transnasal endoscopic choanoplasty should be performed in unilateral CA. The extent of the resection should be large enough so that it is not necessary to insert a stent [13]	I	A	Strongly recommended
20	In the case of bilateral CA, where stents were used for a nasal passage, they should be changed endoscopically several times a week in the first and second postoperative weeks *	IIa	D	Recommended
21	Discharge from the hospital should be planned for the second postoperative week or as soon as possible after surgical treatment of bilateral CA *	IIa	D	Recommended
22, 23	In the case of bilateral CA, stents can be changed on an outpatient basis in the second postoperative week. They can be changed twice in the third week and once from the fourth week onwards *	IIb	D	Optional
24	In the case of bilateral CA, the stent should be removed in the fourth postoperative week, and nasal patency should be reevaluated a week later *	I	D	Strongly recommended
25	Photo documentation of the lumen of the choana should be done for unilateral or bilateral CA *	IIa	D	Recommended
26	A child with bilateral CA should be monitored for at least two years after the procedure or until the end of growth *	I	C	Strongly recommended
27	In case of problems with nasal patency and more than 50% reduction of the choanal lumen, a revision transnasal endoscopic choanoplasty should be performed [13]	IIa	A	Recommended
28	A proton pump inhibitor should be used daily for the first two months after unilateral or bilateral CA surgery [25]	IIa	C	Recommended
29	During the postoperative treatment of the infant, drops of saline solution and nasal glucocorticoid with low systemic absorption should be used daily intranasally for the first two months [13,28]	I	C	Strongly recommended
30	Antibiotic intranasal drops should be used exceptionally in the first postoperative week or later when noticeable purulent discharge appears *	IIa	D	Recommended
31	The first postoperative examination with assessment of nasal breathing with the mouth closed and feeding by mouth without pauses for breathing should be performed on the first postoperative day of bilateral CA treatment *	IIa	D	Recommended
32	On the second postoperative day of treatment for bilateral CA, discharge from the hospital can be planned *	IIb	D	Optional
33	Endoscopy should be performed one week after surgery, two weeks after surgery, and then four weeks after surgery for bilateral CA *	IIa	D	Recommended
34	Discharge from the hospital can be planned on the first postoperative day of treatment for unilateral CA *	IIa	D	Recommended

Nr—number of recommendation; L—level of evidence; Gr–evidence grade; Strength—the strength of recommendation. An explanation of the levels of evidence, evidence grades and determination of the strength of the recommendations are found in Table 4. CA–choanal atresia *—based on own experience.

**Table 3:** Three-step process of reviewing the supporting literature in preparing Slovenian recommendations for the treatment of choanal atresia.

Step of Literature Review: Article Types	Database, a *Search Query*
1. step: systematic reviews, meta-analyses, clinical guidelines, recommendations	Cochrane Library: choan* AND atresia in Title Abstract Keyword–(Word variations have been searched)
	Web of Science: TI = (choan* AND atresia) AND (TI = ((systematic review) OR (meta*) ) OR TS = ((systematic review) OR meta* OR recommend* OR guideline*))
	Scopus: (TITLE(choan* and atresia) AND TITLE-ABS-KEY((systematic AND review) OR (meta*) OR recommend* OR guideline*))
	Pubmed: (choan*[Title] AND atresia[Title]) AND ((systematic review)[Title/Abstract] OR meta*[Title/Abstract] OR recommend *[Title/Abstract] OR guideline*[Title/Abstract])
2. step: randomized controlled clinical studies	Web of Science: TI = (choan* AND atresia) AND TI = (randomi*)
	Pubmed: (choan*[Title] AND atresia[Title]) AND (randomi*[Title/Abstract])
	Scopus: (TITLE(choan* AND atresia) AND TITLE-ABS-KEY(randomi*))
	Literature review from systematic reviews, meta-analyses, clinical guidelines and recommendations included after 1st step of the literature review
3. step: other types of clinical articles	Literature review from studies included after the 1st and 2nd steps of the literature review.

With the first two steps of the supporting literature review, eight systematic reviews or meta-analyses and four randomized controlled clinical trials were searched. * is an operator

**Table 4:** Determination of recommendations’ strength.

Evidence Grade	Type of Studies	The Preponderance of Benefit or Harm	Balance of Benefit and Harm
A (high-quality)	the data are derived from consistent level 1 studies	Strongly recommended	Optional
B (moderate quality)	the data are derived from consistent level 2 or 3 studies or extrapolations from level 1 studies	Recommended or strongly recommended	Optional
C (low quality)	the data are derived from level 4 studies or extrapolations from level 2 or 3 studies	Recommended	Optional
D (very low quality)	the data are derived from level 5 evidence or troublingly inconsistent or inconclusive studies of any level	Optional	No recommendation

## Data Availability

Not applicable.
